# Use of CPAP Failure Score to Predict the Risk of Helmet-CPAP Support Failure in COVID-19 Patients: A Retrospective Study

**DOI:** 10.3390/jcm11092593

**Published:** 2022-05-05

**Authors:** Francesco Alessandri, Antonella Tosi, Francesco De Lazzaro, Chiara Andreoli, Andrea Cicchinelli, Cosima Carrieri, Quirino Lai, Francesco Pugliese, on behalf of the Policlinico Umberto I COVID-19 Group

**Affiliations:** 1 Department of General Surgery and Organ Transplantation Unit, Sapienza University of Rome, Policlinico Umberto I, 00161 Rome, Italy; quirino.lai@uniroma1.it (Q.L.); f.pugliese@uniroma1.it (F.P.); 2 Department of Anesthesiology, Critical Care and Pain Medicine, Sapienza University of Rome, Policlinico Umberto I, 00161 Rome, Italy; antonella.tosi1@gmail.com (A.T.); franzdelazzaro@gmail.com (F.D.L.); andreacicchinelli@gmail.com (A.C.); cosima.carrieri@gmail.com (C.C.); 3 Department of Radiology, Sapienza University of Rome, Policlinico Umberto I, 00161 Rome, Italy; chiaraandreoli@gmail.com

**Keywords:** continuous positive airway pressure, non-invasive ventilation, respiratory failure, severe acute respiratory syndrome coronavirus 2

## Abstract

(1) Background: the aim of this study was to create a score to predict the incidence of CPAP failure in COVID-19 patients early. (2) Methods: in this retrospective observational study, we included all consecutive adult patients admitted between February and April 2021. The main outcome was the failure of CPAP support (intubation or death). (3) Results: two-hundred and sixty-three COVID-19 patients were managed with CPAP. The population was divided in short-CPAP (CPAP days ≤ 10; 72.6%) and long-CPAP (>10; 27.4%) groups. After balancing the entire population using a stabilized IPTW method, we applied a multivariable logistic regression analysis to identify the risk factors for CPAP failure. We used the identified covariates to create a mathematical model, the CPAP Failure Score (CPAP-FS). The multivariable logistic regression analysis identified four variables: SpO_2_ (OR = 0.86; *p*-value = 0.001), P/F ratio (OR = 0.99; *p*-value = 0.008), the Call Score (OR = 1.44; *p*-value = 0.02), and a pre-existing chronic lung disease (OR = 3.08; *p*-value = 0.057). The beta-coefficients obtained were used to develop the CPAP-FS, whose diagnostic ability outperformed other relevant COVID-19-related parameters (AUC = 0.87; *p*-value < 0.0001). We validated the CPAP-FS using a 10-fold internal cross-validation method which confirmed the observed results (AUCs 0.76–0.80; *p*-values < 0.0001). (4) Conclusions: the CPAP-FS can early identify COVID-19 patients who are at risk of CPAP failure.

## 1. Introduction

Patients affected by severe acute respiratory syndrome coronavirus 2 (SARS-CoV2) infection are asymptomatic or show mild symptoms in about 80% of cases. However, 15% of patients develop severe acute respiratory failure, and 5% of critical patients are admitted to the intensive care unit (ICU) [1]. Optimal respiratory management to improve hypoxia and to preserve the lungs includes adequate mechanical ventilation, timing and duration of respiratory support, and ventilation mode. Strategies are controversial, and evidence is rapidly evolving [2,3,4]. To prevent self-induced lung injury in severe coronavirus disease 2019 (COVID-19) pneumonia, initial recommendations suggested early intubation and invasive mechanical ventilation. However, the time from ICU admission to intubation was not associated with increased survival in patients with COVID-19 [5,6,7]. Furthermore, COVID-19 patients who were treated with non-invasive ventilation (NIV) in the ICU were burdened by a 2-fold higher risk of failure than patients affected by non-COVID-19 acute hypoxemic respiratory failure [8].

During the recent COVID-19 pandemic, to address the dramatic bed saturation in the ICU, respiratory support with helmet continuous positive airway pressure (CPAP) has been used to treat patients with less severe respiratory failure outside the ICU [9]. The physiological rationale for a positive end-expiratory pressure (PEEP), delivered by a non-invasive device, has been discussed from the beginning of the pandemic, but the prompt recognition of patients at risk for non-invasive respiratory support failure remains challenging [10]. Several scores have been proposed to predict the optimal timing for intubation in patients affected by acute hypoxic respiratory failure, yet none of them have been validated in COVID-19 [11,12,13].

The aim of this study is to create a score that could predict the incidence of CPAP failure in COVID-19 patients at the time of hospital presentation with respiratory compromise (CPAP Failure Score—CPAP-FS).

## 2. Materials and Methods

This retrospective observational study was approved by the Local Ethics Board of Policlinico Umberto I, Rome, Italy (N°109/2020). Between February and April 2021, we included all consecutive adult patients (≥18 years) admitted to the Policlinico Umberto I of Rome for bilateral interstitial pneumonia due to SARS-CoV-2 infection, treated with helmet-CPAP during the hospital stay. The population has been evaluated by reviewing electronic health records of prospectively collected data. Exclusion criteria were an age < 18 years, support with NIV different from helmet-CPAP, and patients still hospitalized in ICU at the time of data analysis. The last follow-up date was 31 May 2021. All patients were positive to polymerase chain reaction testing of a nasopharyngeal sample for SARS-CoV-2. Helmet-CPAP was considered in patients with arterial oxygen pressure (PaO_2_) < 60 mmHg and/or respiratory rate (RR) > 30/min on maximal oxygenation therapy for 15 min (Venturi mask with oxygen flow 15 L/min). Criteria for intubation were persistent or worsening acute respiratory failure (SpO_2_ < 88%, RR > 30/min) despite CPAP set to FiO_2_ 100% and PEEP 10 cm H_2_O. The decision not to intubate after CPAP failure was made by the treating physician.

The main outcome of the study was the failure of CPAP support. CPAP failure was defined as a composite variable comprehending: the need for post-CPAP orotracheal intubation, and/or death during the hospital stay.

The Strengthening the Reporting of Observational Studies in Epidemiology (STROBE) guidelines were followed to create the study. The guarantor of the data quality was the Data Manager of the Study Group (F.A.). Errors and missing data were identified throughout the database and solved, when possible, with specific queries. A detailed table reporting the missing data present in the database is shown in the Supplementary Materials (Table S1).

### Statistical Analysis

Continuous variables were reported as medians and interquartile ranges (IQR). Categorical variables were described as numbers and percentages. Comparisons between groups were made using Fisher’s exact test or chi-squared test for categorical variables, as appropriate. Mann–Whitney was used for continuous variables. Missing data relative to study covariates (Table S1) always involved less than 10% of patients. In all the cases, missing data were handled with a single imputation method. In detail, a median of nearby points imputation was adopted. The median instead of the mean was adopted due to the skewed distribution of the managed variables [14]. 

The entire population was preliminarily divided in two groups according to the length of CPAP support.

We identified the best cut-off to divide the population using the Receiver Operating Characteristic (ROC) analysis. As specified in the data reported in Table S2, different cut-offs for the risk of CPAP failure were tested. The Youden’s index was used to evaluate the best diagnostic accuracy of the threshold identified. We selected as CPAP failure cut-off the value of 10 days (approximately corresponding to the 70th centile of the population) due to its higher Youden’s index value. With the intent to compensate for the non-randomized design of this retrospective study, the two groups were “balanced” using a stabilized inverse probability therapy weighting (IPTW). We generated a propensity score for each patient on the original population of 263 patients. The score was created using a multivariate logistic regression model considering CPAP failure (no vs. yes) as the dependent variable. We selected 20 possible clinically relevant confounders as covariates: COVID-19 first wave, age, male sex, arterial hypertension, type-2 diabetes mellitus, cardiovascular comorbidity, liver comorbidity, asthma, chronic lung disease, renal comorbidity, neurological comorbidity, obesity, HIV or malignancy, need for ICU stay, CT scan lung damage %, P/F ratio, Call Score, SpO_2_, C-reactive protein, and D-dimer. 

All the covariates were available at the beginning of CPAP support to avoid the risk of a possible immortal time bias in covariate selection. With the intent to reduce the artificial increase of the sample size, and, therefore, of the type I error rate (i.e., the increased number of false positives) caused by the inflated sample size in the pseudo data, we used stabilized weights (SW) according to the formula:

SW = *p*/PS for the study group, and SW = (1 − *p*)/(1 − PS) for the control group (1)

where *p* is the probability of etiology without considering covariates, and PS is the propensity score [15].

Because *p*-values can be biased by population size, results from the comparisons between covariates subgroups were reported as effect size (Cohen’s D value): values lower than |0.1| indicated very small differences between means, values between |0.1| and |0.3| indicated small differences, values between |0.3| and |0.5| indicated moderate differences, and values greater than |0.5| indicated considerable differences [16].

A multivariable logistic regression analysis was conducted in the post-IPTW population to identify the risk factors for CPAP failure. The same 20 covariates used to calculate the IPTW were investigated. The best model was calculated selecting the most statistically significant covariates with a backward conditional approach. An adjustment of the model for age and sex was done. Odds ratios (OR) and 95.0% confidence intervals (95.0% CI) were reported for significant variables. OR and 95.0% CI were based on 1000 bootstrap samples. 

Using the beta-coefficients of the identified covariates, a mathematical model was created, namely the CPAP-FS. 

The diagnostic ability of the proposed score was compared with other clinically relevant parameters using the c-statistical analysis. Area under the curve (AUC) and 95.0% CI were reported for significant variables. The analyses were performed in the post-IPTW pseudo population (used to obtain the score) and validated in the initial pre-IPTW unbalanced population.

With the intent to further validate the observed results, a 10-fold internal cross-validation method was adopted, randomly splitting the original pre-IPTW samples of 263 cases into 10 equally-sized (*n* = 200) sub-samples, respectively. CPAP-FS was tested again in each sub-group in terms of prognostic ability.

Survival analyses were performed using the Kaplan–Meier method, and the log-rank test was adopted to compare the obtained survivals.

Variables with a *p* < 0.05 were considered statistically significant. Statistical analyses and plots were run using the SPSS statistical package version 27.0 (SPSS Inc., Chicago, IL, USA).

## 3. Results

Between February and April 2021, 263 hospitalized COVID-19 patients were managed with CPAP support at Policlinico Umberto I of Rome. The characteristics and the severity of COVID-19 are reported in Table 1. The entire population was divided into two groups: short-CPAP (CPAP days ≤ 10; *n* = 191, 72.6%) and long-CPAP (CPAP days > 10; *n* = 72, 27.4%). Patients of the two groups were similar for comorbidities such as arterial hypertension, diabetes, asthma, and chronic lung disease. Age and gender were not significantly different in the two groups. The two groups were also similar comparing several radiological, clinical, and biochemical parameters of COVID-19 severity. As an example, the median percentage of pulmonary damage evaluated by CT scan, the P/F ratio, and the C-reactive protein were not statistically relevant. The SpO_2_ recorded at hospital admission was lower in the short-CPAP group (93% vs. 95%; *p*-value = 0.02), indicating a potentially more severe COVID-19 pneumonia in this group. The incidence of ICU admission was higher in the short-CPAP group compared to the long-CPAP group (29.3 vs. 11.1%; *p*-value = 0.002). Furthermore, the short-CPAP group had a higher rate of intubation (41/43, 95.3%; *p*-value < 0.0001). As expected, the median hospital stay was longer in the long-CPAP group than in the short-CPAP group (31 vs. 16 days; *p*-value < 0.0001).

### 3.1. Stabilized IPTW Effect

With the intent to minimize the effect of selection biases caused by the non-randomized design of this retrospective study, the entire population was “artificially” balanced using a stabilized IPTW method. As reported in Table 2, the population was efficaciously “balanced” for the 20 potential confounders adopted. In detail, before the IPTW, 10 variables showed very small differences, seven showed small differences, two showed moderate differences, and one variable showed a considerable difference. After the IPTW, 12 variables showed very small differences, and eight showed small differences. Despite the stabilized IPTW being adopted with the intent to minimize the potential reduction of the sample size of the initial population, the post-IPTW pseudo population reduced to 168 cases.

### 3.2. CPAP-FS

A multivariable logistic regression model was performed on the post-IPTW population with the intent to identify the risk factors for CPAP failure. As reported in Table 3 three variables measured at hospital admission were independent factors to predict the risk of CPAP failure: SpO_2_ (OR = 0.86; *p*-value = 0.001), P/F ratio (OR = 0.99; *p*-value = 0.008), and the Call Score (OR = 1.44; *p*-value = 0.02). A preexisting chronic lung disease only neared statistical relevance (OR = 3.08; *p*-value = 0.057).

According to the obtained beta-coefficients, we proposed the CPAP Failure Score: 

7.315 + 0.512 (if male) + 0.044 × age + 1.124 (if chronic lung disease) + 0.365 × Call Score *−* 0.153 × SpO_2_ − 0.008 × P/F ratio. Table 4 shows the characteristics of the population divided into two groups: CPAP failure and no CPAP failure

### 3.3. Diagnostic Ability

The CPAP-FS was superior to other relevant COVID-19-related parameters (patient age, D-dimer, P/F ratio, Call Score, SpO_2_, and the presence of comorbidities or chronic lung disease), with an AUC = 0.87 (*p*-value < 0.0001) (Table 5).

The score was further validated exploring its diagnostic ability in the entire pre-IPTW unbalanced population of 263 cases. Moreover, in this case, the CPAP-FS had a better diagnostic ability when compared to other parameters connected to COVID-19 severity (AUC = 0.78, *p*-value < 0.0001).

In addition, we performed an internal validation of the score. In the 10-fold internal validation, the CPAP-FS was always superior to all the other variables in terms of diagnostic ability (AUCs 0.76–0.80, *p*-values < 0.0001) (Table 6).

### 3.4. CPAP-FS and CPAP Use

Stratifying the entire population in four quartiles (Table S3), we identified four different sub-classes showing significantly different rates of CPAP failure and a different temporal distribution of the risk of failure.

**Figure 1:**
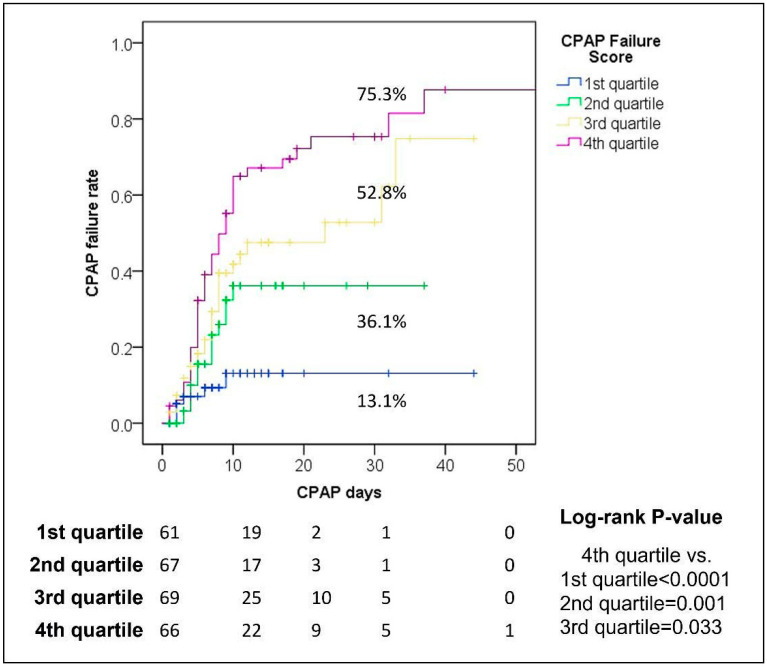
Different CPAP failure rates in the different sub-classes of CPAP Failure Score.

In detail, the patients within the first quartile of the score (value ≤ −2.90) presented a low one-month risk of CPAP failure (13.1%). In the second quartile (value −2.90–−1.61), the failure rate was 36.1%. Finally, in the third (value −1.60–−0.11) and fourth quartile (value ≥ −0.10), the failure rates were higher, respectively, 52.8 and 75.3% (Figure 1 and Table S4).

**Figure 2:**
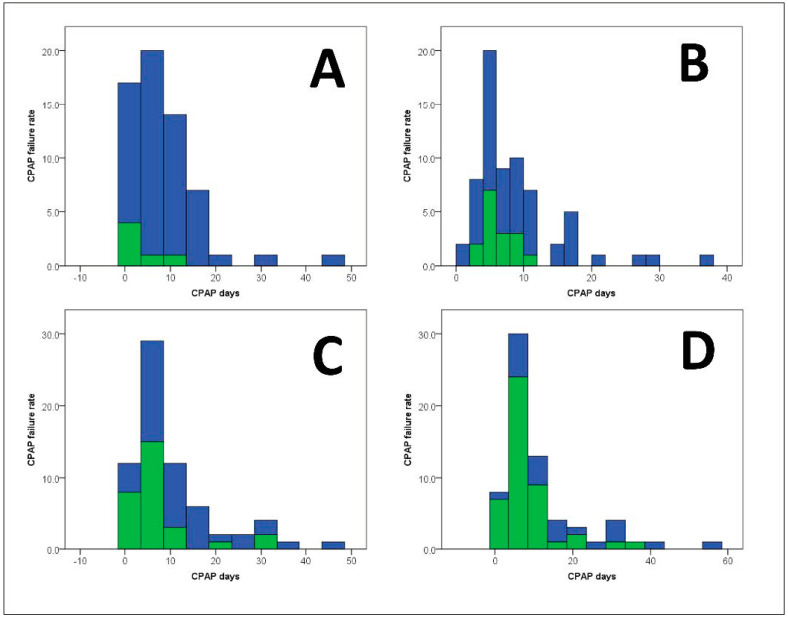
Distribution of CPAP failures in the different sub-classes of CPAP Failure Score. (**A**) First quartile; (**B**) second quartile; (**C**) third quartile; (**D**) fourth quartile. Green = failure; blue = no failure.

Interestingly, the patients belonging to the first and second quartiles developed CPAP failure predominantly during the first 10 days of CPAP support (*n* = 25). After this period, the incidence of CPAP failure was only anecdotic (*n* = 3). Conversely, in the third and fourth quartile patients, the number of failures was consistent not only during the first 10 days, but also after day-20 of non-invasive support (Figure 2).

## 4. Discussion

Data from this retrospective observational study suggest that CPAP-FS predicts the incidence of CPAP failure in COVID-19 patients affected by acute hypoxemic respiratory failure and treated with helmet-CPAP. The severity of the score correlates with the risk of failure, and stratifies its temporal distribution. Patients with a lower CPAP-FS (first and second quartile) have a higher risk of failure within the first 10 days of CPAP, whereas more severe patients (third and fourth quartile) show an elevated risk of failure even beyond 10 days of treatment (especially after 20 days).

All COVID-19 patients enrolled in the study received non-invasive support through helmet-CPAP. Over the years, this device has shown several advantages in the management of severe acute respiratory failure compared to other non-invasive devices. It provides a constant and stable PEEP delivered by a free-flow system and a PEEP valve [17] without the need for a ventilator, a crucial characteristic for its use in non-ICU settings [18]. Helmet-CPAP has shown a lower risk of environmental contamination and nosocomial transmission of infections thanks to a lower level of leaks than nasal and face masks [19]. Furthermore, the use of a face mask interface tends to be less-tolerated, leading to a high failure rate and the need for intubation and invasive mechanical ventilation [20]. These advantages have led to a better outcome in recent studies when helmet-CPAP was compared to other non-invasive respiratory devices. In a metanalysis by Ferreyro et al., respiratory support with helmet-CPAP was compared to high-flow nasal cannula (HFNC) and NIV delivered by face mask, leading to a lower risk of endotracheal intubation and death with the first device [21]. In addition, regarding the management of COVID-19 respiratory failure, the secondary outcomes of the HENIVOT randomized controlled trial showed that the use of helmet NIV, when compared to HFNC, led to a significantly lower incidence of intubation, and a higher number of invasive mechanical-ventilation-free days at 28 days [22].

Many multiparametric scores have been designed and validated to predict the risk of intubation and invasive mechanical ventilation in ICU patients. Of these, only a few can be used specifically to evaluate the risk of NIV failure in COVID-19 patients. In a previous retrospective study by Liu et al., an online calculator was validated to predict non-invasive respiratory support failure in a cohort of 652 COVID-19 patients [13]. Among those, only 286 patients were treated with NIV; the remaining 366 patients received HFNC. The variables used to develop this nomogram were age, number of comorbidities, ROX index ((SpO_2_/FiO_2_)/respiratory rate), Glasgow coma scale, and the use of vasopressors during the first day of NIV support.

Compared to the CPAP-FS, the nomogram proposed by Liu et al. includes patients managed with devices other than helmet-CPAP. Most of the patients were treated with HFNC, which has shown poorer important secondary outcomes in the recent HENIVOT trial on COVID-19 patients [22]. 

The HACOR score, previously used to predict NIV failure in hypoxemic patients, has been evaluated in COVID-19 by Guia et al. [23]. It is a bedside scoring system including five parameters: heart rate, acidosis, consciousness, oxygenation, and respiratory rate. Guia et al. demonstrated that after one hour of NIV, a score > 5 predicted NIV failure in COVID-19 patients with a diagnostic accuracy of 82%. Unlike the HACOR score that considered parameters recorded before NIV initiation, the CPAP-FS can be administered at hospital admission, and combines general and respiratory variables with the CALL score, a measure of disease progression [24]. Furthermore, the diagnostic ability of the CPAP-FS is higher than the accuracy of other parameters typically associated with COVID-19 severity, and the IPTW balancing has further validated the score, mitigating the potential bias due to the retrospective nature of the study. Our analysis demonstrates that CPAP-FS can be used to identify patients who benefit from non-invasive treatment early, and distinguish them from those who have a greater risk of failure. This could be extremely useful during times of resource constraint, such as COVID pandemic waves, when a considerable number of patients with severe acute respiratory failure present to the Emergency Department [25]. This score could help allocate patients to either the ICU or the medical ward based on the risk stratification. More specifically, patients who have a lower risk of CPAP failure could be safely managed in a non-ICU setting. On the other hand, patients with a high CPAP-FS prompt a stricter monitoring, and could be considered for early intubation [4]. Therefore, early identification of CPAP failure is a promising strategy to improve outcome. Patients presenting with a low CPAP-FS have a low risk of CPAP failure within the first 10 days of the support; past this cut-off, the risk becomes negligible. In these patients, respiratory support with helmet-CPAP is particularly indicated to overcome the virus-induced acute pulmonary insult. Patients belonging to the more severe classes show an elevated risk of failure after 20 days from the introduction of CPAP. This could probably be associated with the onset of complications related to the prolonged treatment, such as superinfections or self-inflicted lung injury [26,27].

This study has some limitations. First, it is retrospective monocentric research. Despite the statistical analysis being designed to minimize the bias, a prospective study is needed to confirm the reliability of the CPAP-FS. Second, adding other specific parameters might further improve the sensitivity and specificity of the score. We considered pulmonary CT scan involvement at hospital admission, but we found it unreliable in predicting CPAP failure, probably because it is a static parameter unable to capture the evolution of the disease. Third, the retrospective nature of the study limited our ability to collect data necessary to calculate other predictive models of CPAP failure. Therefore, we could not compare the CPAP-FS with other available scores.

## 5. Conclusions

During the recent pandemic, the treatment of acute hypoxemic respiratory failure associated with COVID-19 has been an incredible challenge for clinicians and healthcare systems because of the overwhelming number of patients requiring respiratory support. CPAP-FS can be an easy tool to identify COVID-19 patients presenting with respiratory compromise who are at risk of CPAP failure early. Nevertheless, prospective studies are needed to better identify the cohort of COVID-19 patients who can benefit from CPAP support.

## Figures and Tables

**Table 1:** Characteristics of the COVID-19 population treated with CPAP.

Variable	Entire Population (N = 263, 100.0%)	Short-CPAP (N = 191, 72.6%)	Long-CPAP (N = 72, 27.4%)	*p*-Value
	Median (IQR) or N (%)			
COVID-19 first wave	89 (33.8)	68 (35.6)	21 (29.2)	0.38
Age, years	72 (62–81)	72 (61–80)	71 (62–83)	0.68
Male sex	176 (66.9)	127 (66.5)	49 (68.1)	0.88
Arterial hypertension	65 (24.7)	53 (27.7)	12 (16.7)	0.08
T2DM	51 (19.4)	33 (17.3)	18 (25.0)	0.17
Cardiovascular comorbidity	59 (22.4)	45 (23.6)	14 (19.4)	0.51
Liver comorbidity	6 (2.3)	5 (2.6)	1 (1.4)	1.00
Asthma	6 (2.3)	6 (3.1)	0 (0.0)	0.19
Chronic lung disease	31 (11.8)	19 (9.9)	12 (16.7)	0.14
Renal comorbidity	19 (7.2)	13 (6.8)	6 (8.3)	0.79
Neurological comorbidity	33 (12.5)	25 (13.1)	8 (11.1)	0.84
Obesity	18 (6.8)	12 (6.3)	6 (8.3)	0.59
HIV or malignancy	10 (3.8)	8 (4.2)	2 (2.8)	0.73
Any comorbidity	172 (65.4)	124 (64.9)	48 (66.7)	0.89
Hospital stay, days	19 (11–30)	16 (10–24)	31 (20–37)	<0.0001
Need for ICU stay	64 (24.3)	56 (29.3)	8 (11.1)	0.002
CPAP use days	7 (4–11)	5 (4–8)	17 (13–27)	<0.0001
CT scan lungs mean damage %	33 (20–50)	33 (18–55)	33 (20–45)	0.42
P/F ratio	242 (177–290)	242 (178–290)	242 (170–295)	0.80
Lymphocytes, 10^3^ cells/μL	0.75 (0.51–1.10)	0.76 (0.53–1.10)	0.74 (0.50–1.11)	0.72
LDH, mU/mL	366 (291–452)	366 (283–453)	364 (294–444)	0.68
Call Score	10 (8–12)	10 (8–12)	10 (9–12)	0.42
SpO_2_	94 (90–96)	93 (89–95)	95 (91–97)	0.02
C-reactive protein, mmol/L	8195 (1487–48,000)	8195 (1438–48,000)	9018 (1737–49,350)	0.59
D-dimer, ng/mL	1089 (633–2140)	1089 (627–1858)	1132 (638–3085)	0.44
Orotracheal intubation	43 (16.3)	41 (21.5)	2 (2.8)	<0.0001
Death	92 (35.0)	81 (42.4)	11 (15.3)	<0.0001
CPAP failure (intubation and/or death)	96 (36.5)	85 (44.5)	11 (15.3)	<0.0001

Abbreviations: CPAP, continuous positive airway pressure; IQR, interquartile ranges; COVID-19, coronavirus disease 19; T2DM, type 2 diabetes mellitus; HIV, human immunodeficiency virus; ICU, intensive care unit; CT, computed tomography; P/F, partial pressure of oxygen in arterial blood/fraction of inspired oxygen; LDH, lactate dehydrogenase; SpO_2_, peripheral capillary oxygen saturation.

**Table 2:** Effect of stabilized IPTW in the population on the variables used to balance the two populations.

Variables	Pre-IPTW			Post-IPTW		
	Short-CPAP (N = 191)	Long-CPAP (N = 72)	Cohen’s D-Value	Short-CPAP (N = 97)	Long-CPAP (N = 71)	Cohen’s D-Value
	Mean ± SD			Mean ± SD		
COVID-19 first wave	0.36 ± 0.48	0.29 ± 0.46	0.14	0.34 ± 0.48	0.30 ± 0.46	0.09
Age	70.27 ± 13.40	70.92 ± 13.72	−0.05	70.64 ± 13.43	69.81 ± 14.28	0.06
Male sex	0.66 ± 0.47	0.68 ± 0.47	−0.03	0.67 ± 0.47	0.59 ± 0.50	0.16
Arterial hypertension	0.28 ± 0.45	0.17 ± 0.38	0.28	0.25 ± 0.43	0.20 ± 0.41	0.10
T2DM	0.17 ± 0.38	0.25 ± 0.44	−0.18	0.21 ± 0.41	0.18 ± 0.39	0.06
Cardiovascular disease	0.24 ± 0.43	0.19 ± 0.40	0.10	0.22 ± 0.42	0.17 ± 0.38	0.13
Liver disease	0.03 ± 0.16	0.01 ± 0.12	0.09	0.02 ± 0.15	0.02 ± 0.12	0.05
Asthma	0.03 ± 0.17	0.00 ± 0.00	0.34	0.02 ± 0.15	0.00 ± 0.00	0.23
Chronic lung disease	0.10 ± 0.30	0.17 ± 0.38	−0.19	0.12 ± 0.33	0.12 ± 0.33	0.01
Renal comorbidity	0.07 ± 0.25	0.08 ± 0.28	−0.06	0.07 ± 0.26	0.07 ± 0.27	−0.01
Neurological disease	0.13 ± 0.34	0.11 ± 0.32	0.06	0.13 ± 0.33	0.11 ± 0.31	0.05
Obesity	0.06 ± 0.24	0.08 ± 0.28	−0.08	0.06 ± 0.23	0.04 ± 0.19	0.10
HIV or malignancy	0.04 ± 0.20	0.03 ± 0.17	0.08	0.04 ± 0.19	0.03 ± 0.17	0.05
Need for ICU stay	0.29 ± 0.46	0.11 ± 0.32	0.51	0.24 ± 0.43	0.27 ± 0.45	−0.07
CT scan lung damage %	36.69 ± 22.80	33.59 ± 20.11	0.15	36.09 ± 22.41	32.06 ± 20.28	0.19
P/F ratio	236.84 ± 79.59	240.42 ± 82.51	−0.04	237.87 ± 79.29	232.38 ± 80.31	0.07
Call Score	9.88 ± 2.08	10.06 ± 2.23	−0.08	9.94 ± 2.08	9.50 ± 2.35	0.20
SpO_2_	91.84 ± 5.29	92.93 ± 5.86	−0.19	92.06 ± 5.12	90.34 ± 9.56	0.21
C-reactive protein	40,073.96 ± 86,244.73	35,689.07 ± 59,617.40	0.06	38,824.67 ± 83,007.51	36,246.87 ± 62,108.37	0.04
D-dimer	1742.88 ± 1939.86	3839.19 ± 10,909.58	−0.22	2007.53 ± 2622.81	2385.01 ± 6264.52	−0.07

Abbreviations: IPTW, inverse probability therapy weighting; CPAP, continuous positive airway pressure; SD, standard deviation; COVID-19, coronavirus disease 19; T2DM, type 2 diabetes mellitus; HIV, human immunodeficiency virus; ICU, intensive care unit; CT, computed tomography; P/F, partial pressure of oxygen in arterial blood/fraction of inspired oxygen; SpO_2_, peripheral capillary oxygen saturation.

**Table 3:** Multivariable logistic regression for the risk of CPAP failure. Post-IPTW population.

Variables	Beta	SE	Wald	OR	95.0% CI		*p*-Value
					Lower	Upper	
SpO_2_	−0.153	0.050	3.17	0.86	0.79	0.93	0.001
P/F ratio	−0.008	0.004	3.41	0.99	0.985	0.998	0.008
Call Score	0.365	0.189	0.99	1.44	1.09	1.91	0.02
Chronic lung disease	1.124	0.995	5.79	3.08	0.93	10.14	0.057
Age	0.044	0.031	6.54	1.05	0.99	1.10	0.10
Male sex	0.512	0.598	13.00	1.67	0.61	4.59	0.33
Constant	7.315	4.721	3.01	1502.64	-	-	0.051
−2Log likelihood: 119.81; Hosmer-Lemeshow Test: 0.97							
Calculation of the CPAP Failure Score 7.315 + 0.512 (if male) + 0.044 × age + 1.124 (if chronic lung disease) + 0.365 × Call Score − 0.153 × SpO_2_ − 0.008 × P/F ratio							

Abbreviations: SE, standard error; OR, odds ratio; 95.0% CI, 95.0% confidence intervals; SpO_2_, peripheral oxygen saturation; P/F, partial pressure of oxygen in arterial blood/fraction of inspired oxygen.

**Table 4:** Characteristics of the COVID-19 population treated with CPAP: comparison between CPAP failure and no CPAP failure Groups.

Variable	No CPAP Failure (*n* = 167, 63.5%)	CPAP Failure (*n* = 96, 36.5%)	*p*-Value
	Median (IQR) or *n* (%)		
COVID-19 first wave	53 (31.7)	36 (37.5)	0.35
Age, years	70 (57–78)	75 (68–84)	<0.0001
Male sex	106 (63.5)	70 (72.9)	0.14
Arterial hypertension	37 (22.2)	28 (29.2)	0.24
T2DM	27 (16.2)	24 (25.0)	0.11
Cardiovascular comorbidity	24 (14.4)	35 (36.5)	<0.0001
Liver comorbidity	3 (1.8)	3 (3.1)	0.67
Asthma	4 (2.4)	2 (2.1)	1.00
Chronic lung disease	15 (9.0)	16 (16.7)	0.08
Renal comorbidity	11 (6.6)	8 (8.3)	0.63
Neurological comorbidity	16 (9.6)	17 (17.7)	0.04
Obesity	13 (7.8)	5 (5.2)	0.61
HIV or malignancy	4 (2.4)	6 (6.3)	0.18
Any comorbidity	93 (55.7)	79 (82.3)	<0.0001
Hospital stay, days	23 (17–33)	11 (7–20)	<0.0001
Need for ICU stay	20 (12.0)	44 (45.8)	<0.0001
CPAP lenght days	8 (5–14)	6 (4–9)	0.001
CT scan lungs mean damage %	30 (20–45)	38 (18–64)	0.02
p/f ratio	252 (210–310)	224 (131–243)	<0.0001
Lymphocytes, 10^3^ cells/μL	0.77 (0.53–1.08)	0.74 (0.49–1.16)	0.81
LDH, mU/mL	366 (295–431)	376 (274–500)	0.26
Call Score	9 (8–12)	11 (10–12)	<0.0001
SpO_2_	94 (91–96)	91 (87–95)	<0.0001
C-reactive protein, mmol/L	8195 (2000–50,100)	8142 (1276–47,075)	0.35
D-dimer, ng/mL	958 (552–1871)	1264 (844–2913)	0.007
Orotracheal intubation	0 (-)	43 (44.8)	<0.0001
Death	0 (-)	92 (95.8)	<0.0001
CPAP failure (intubation and/or death)	0 (-)	96 (100.0)	<0.0001

Abbreviations: CPAP, continuous positive airway pressure; IQR, interquartile ranges; COVID-19, coronavirus disease 19; T2DM, type 2 diabetes mellitus; HIV, human immunodeficiency virus; ICU, intensive care unit; CT, computed tomography; p/f, partial pressure of oxygen in arterial blood/fraction of inspired oxygen; LDH, lactate dehydrogenase; SpO_2_, peripheral capillary oxygen saturation.

**Table 5:** Diagnostic ability of the CPAP Failure Score compared to other relevant clinical factors for the potential failure of CPAP support: validation of the model in the pre- and post-IPTW population.

	Post-IPTW (N = 168)					Pre-IPTW (N = 263)				
Variable	AUC	SE	95.0% CI		*p*-Value	AUC	SE	95.0% CI		*p*-Value
CPAP Failure Score	0.87	0.03	0.81	0.93	<0.0001	0.78	0.03	0.72	0.83	<0.0001
Age	0.77	0.04	0.69	0.85	<0.0001	0.66	0.03	0.59	0.73	<0.0001
D-dimer	0.73	0.05	0.64	0.82	<0.0001	0.60	0.04	0.53	0.67	0.007
1-(P/F ratio)	0.71	0.04	0.62	0.79	<0.0001	0.68	0.04	0.61	0.74	<0.0001
Call Score	0.69	0.04	0.60	0.79	<0.0001	0.68	0.03	0.61	0.74	<0.0001
1-(SpO_2_)	0.69	0.05	0.59	0.79	<0.0001	0.65	0.04	0.58	0.72	<0.0001
Comorbidity	0.63	0.05	0.53	0.72	0.01	0.63	0.04	0.57	0.70	<0.0001
Chronic lung disease	0.59	0.05	0.49	0.69	0.08	0.54	0.04	0.47	0.61	0.30
C-reactive protein	0.43	0.05	0.33	0.53	0.15	0.47	0.04	0.39	0.54	0.35
Male sex	0.45	0.05	0.35	0.55	0.31	0.55	0.04	0.48	0.62	0.20
CT scan lung damage %	0.53	0.06	0.41	0.65	0.52	0.59	0.04	0.51	0.66	0.02

Abbreviations: IPTW, inverse probability therapy weighting; AUC, area under the curve; SE, standard error; 95.0% CI, 95.0% confidence intervals; CPAP, continuous positive airway pressure; SpO_2_, peripheral capillary oxygen saturation; P/F, partial pressure of oxygen in arterial blood/fraction of inspired oxygen; CT, computed tomography.

**Table 6:** Diagnostic ability of the CPAP Failure Score compared with other relevant clinical factors for the potential failure of CPAP approach: 10-fold internal validation of the model in the pre-IPTW population.

	1st (N = 200)		2nd (N = 200)		3rd (N = 200)		4th (N = 200)		5th (N = 200)		6th (N = 200)		7th (N = 200)		8th (N = 200)		9th (N = 200)		10th (N = 200)	
Variable	AUC	P	AUC	P	AUC	P	AUC	P	AUC	P	AUC	P	AUC	P	AUC	P	AUC	P	AUC	P
CPAP Failure Score	0.80	<0.0001	0.76	<0.0001	0.77	<0.0001	0.76	<0.0001	0.77	<0.0001	0.77	<0.0001	0.80	<0.0001	0.77	<0.0001	0.78	<0.0001	0.77	<0.0001
Age	0.71	<0.0001	0.64	0.002	0.65	0.001	0.68	<0.0001	0.64	0.001	0.66	<0.0001	0.67	<0.0001	0.64	0.001	0.66	<0.0001	0.67	<0.0001
D-dimer	0.60	0.02	0.63	0.003	0.61	0.01	0.57	0.12	0.62	0.006	0.60	0.02	0.63	0.004	0.62	0.003	0.61	0.01	0.61	0.01
1-(p/f ratio)	0.68	<0.0001	0.65	<0.0001	0.68	<0.0001	0.66	<0.0001	0.65	<0.0001	0.68	<0.0001	0.69	<0.0001	0.69	<0.0001	0.67	<0.0001	0.68	<0.0001
Call Score	0.69	<0.0001	0.68	<0.0001	0.66	<0.0001	0.65	<0.0001	0.66	<0.0001	0.66	<0.0001	0.69	<0.0001	0.67	<0.0001	0.68	<0.0001	0.69	<0.0001
1-(SpO_2_)	0.66	<0.0001	0.65	<0.0001	0.68	<0.0001	0.64	0.001	0.64	0.001	0.63	0.003	0.67	<0.0001	0.67	<0.0001	0.64	0.001	0.63	0.002
Comorbidity	0.64	0.001	0.64	0.002	0.63	0.002	0.62	0.006	0.64	0.001	0.60	0.03	0.65	0.001	0.63	0.002	0.63	0.002	0.63	0.003
Chronic lung disease	0.56	0.16	0.54	0.37	0.55	0.22	0.53	0.45	0.55	0.23	0.53	0.55	0.56	0.15	0.55	0.26	0.55	0.26	0.53	0.47
C-reactive protein	0.46	0.37	0.48	0.65	0.45	0.24	0.47	0.50	0.48	0.65	0.48	0.57	0.43	0.12	0.46	0.34	0.47	0.47	0.47	0.44
Male sex	0.54	0.38	0.54	0.33	0.53	0.47	0.54	0.34	0.57	0.12	0.56	0.19	0.55	0.29	0.53	0.43	0.57	0.10	0.53	0.55
CT scan lungs damage %	0.57	0.12	0.60	0.02	0.56	0.14	0.53	0.47	0.55	0.21	0.60	0.02	0.63	0.004	0.60	0.02	0.61	0.009	0.57	0.11

Abbreviations: IPTW, inverse probability therapy weighting; AUC, area under the curve; SE, standard error; 95.0% CI, 95.0% confidence intervals; CPAP, continuous positive airway pressure; SpO_2_, peripheral capillary oxygen saturation; p/f, partial pressure of oxygen in arterial blood/fraction of inspired oxygen; CT, computed tomography.

## Data Availability

Data available on request due to restrictions e.g., privacy or ethical. The data presented in this study are available on request from the corresponding author. The data are not publicly available due to the lack of a link.

## Supplementary Materials

The following supporting information can be downloaded at: https://www.mdpi.com/article/10.3390/jcm11092593/s1: Table S1: Missing data reported in the cohort of 263 patients treated with CPAP after COVID-19 infection; Table S2: Identification of the best CPAP threshold to use for identifying the risk of CPAP failure after COVID-19 infection; Table S3: Diagnostic ability of the CPAP Failure Score compared to other relevant clinical factors for the potential failure of CPAP support: validation of the model in the pre- and post-IPTW population; Table S4: Stratification of the CPAP-FS in quartiles and deciles.
