# Supplementary material for: Use of CPAP Failure Score to Predict the Risk of Helmet-CPAP Support Failure in COVID-19 Patients: A Retrospective Study
Source: J Clin Med. 2022 May 5;11(9):2593. doi: 10.3390/jcm11092593 (PMC9104739; doi:10.3390/jcm11092593)
Supplement: Supplementary file 1 [file jcm-11-02593-s001.zip › jcm-1653171-supplementary.pdf]

Table S1. Missing data reported in the cohort of 263 patients treated with CPAP after COVID-19 infection.

Variable	Missing data	Percentage
**COVID-19 first wave**	0	0.0
**Age, years**	0	0.0
**Male sex**	0	0.0
**Arterial hypertension**	0	0.0
**T2DM**	0	0.0
**Cardiovascular comorbidity**	0	0.0
**Liver comorbidity**	0	0.0
**Asthma**	0	0.0
**Chronic lung disease**	0	0.0
**Renal comorbidity**	0	0.0
**Neurological comorbidity**	0	0.0
**Obesity**	0	0.0
**HIV or malignancy**	0	0.0
**Hospital stay, days**	0	0.0
**Need for ICU stay**	0	0.0
**Orotracheal intubation**	0	0.0
**Death**	0	0.0
**CPAP use days**	0	0.0
**CT scan damage percentage right lung**	7	2.7
**CT scan damage percentage left lung**	7	2.7
**CT scan mean damage percentage**	7	2.7
**P/F ratio**	25	9.5
**Any comorbidity**	0	0.0
**Lymphocytes, 10^3 cells/μL**	0	0.0
**LDH, mU/mL**	11	4.2
**Call Score**	11	4.2
**SpO2**	15	5.7
**C-reactive protein, mmol/L**	4	1.5
**D-dimer, ng/mL**	5	1.9

Abbreviations: COVID-19, coronavirus disease 19; T2DM, type 2 diabetes mellitus; HIV, human immunodeficiency virus; ICU, intensive care unit; CPAP, continuous positive airway pressure; CT, computed tomography; P/F, partial pressure of oxygen in arterial blood/ fraction of inspired oxygen; LDH, lactate dehydrogenase; SpO2, peripheral capillary oxygen saturation.

Table S2. Identification of the best CPAP threshold to use for identifying the risk of CPAP failure after COVID-19 infection.

CPAP use days	Centile	Sesitivity	Specificity	Youden's index
**2**	10	0.88	0.11	0.02
**4**	20	0.78	0.16	0.06
**5**	30	0.66	0.22	0.12
**6**	40	0.52	0.33	0.15
**7**	50	0.45	0.42	0.13
**8**	60	0.34	0.48	0.18
**10**	70	0.19	0.59	0.23
**13**	80	0.08	0.71	0.20
**20**	90	0.06	0.88	0.06

Abbreviations: CPAP, continuous positive airway pressure.

Table S3. Diagnostic ability of the CPAP Failure Score compared to other relevant clinical factors for the potential failure of CPAP support: validation of the model in the pre- and post-IPTW population.

	Post-IPTW (N=168)					Pre-IPTW (N=263)				
Variable	AUC	SE	95.0%CI		*P*-value	AUC	SE	95.0%CI		*P*-value
**CPAP Failure Score**	0.87	0.03	0.81	0.93	<0.0001	0.78	0.03	0.72	0.83	<0.0001
**Age**	0.77	0.04	0.69	0.85	<0.0001	0.66	0.03	0.59	0.73	<0.0001
**D-dimer**	0.73	0.05	0.64	0.82	<0.0001	0.60	0.04	0.53	0.67	0.007
**1 - (P/F ratio)**	0.71	0.04	0.62	0.79	<0.0001	0.68	0.04	0.61	0.74	<0.0001
**Call Score**	0.69	0.04	0.60	0.79	<0.0001	0.68	0.03	0.61	0.74	<0.0001
**1 - (SpO2)**	0.69	0.05	0.59	0.79	<0.0001	0.65	0.04	0.58	0.72	<0.0001
**Comorbidity**	0.63	0.05	0.53	0.72	0.01	0.63	0.04	0.57	0.70	<0.0001
**Chronic lung disease**	0.59	0.05	0.49	0.69	0.08	0.54	0.04	0.47	0.61	0.30
**C-reactive protein**	0.43	0.05	0.33	0.53	0.15	0.47	0.04	0.39	0.54	0.35
**Male sex**	0.45	0.05	0.35	0.55	0.31	0.55	0.04	0.48	0.62	0.20
**CT scan lung damage %**	0.53	0.06	0.41	0.65	0.52	0.59	0.04	0.51	0.66	0.02

Abbreviations: IPTW, inverse probability therapy weighting; AUC, area under the curve; SE, standard error; 95.0%CI, 95.0% confidence intervals; CPAP, continuous positive airway pressure; SpO2, peripheral capillary oxygen saturation; P/F, partial pressure of oxygen in arterial blood/ fraction of inspired oxygen; CT, computed tomography.

Table S4. Stratification of the CPAP-FS in quartiles and deciles.

CPAP-FS cut-off	CPAP-FS value
1st decile	-4.13
2nd decile	-3.20
1st quartile	-2.90
3rd decile	-2.47
4th decile	-1.98
Median	-1.60
6th decile	-0.91
7th decile	-0.51
3rd quartile	-0.10
8th decile	0.24
9th decile	0.80
